# Correction to “CD14+ Dendritic‐Shaped Cells Functioning as Dendritic Cells in Rheumatoid Arthritis Synovial Tissues”

**DOI:** 10.1002/acr2.11777

**Published:** 2025-01-03

Rie Kurose , Takashi Satoh , Akira Kurose , Yasuyuki Ishibashi , Miwa Uzuki , Yuji Wakai , Tomoyuki Sasaki , Kinji Ishida , Katsutoshi Ogasawara , and Takashi Sawai . CD14+ Dendritic‐Shaped Cells Functioning as Dendritic Cells in Rheumatoid Arthritis Synovial Tissues. ACR Open Rheumatology. 2024;6: 412–420.38638058 10.1002/acr2.11670PMC11246827

In page 414, Line 3; “20 mL”, Line 5; “5 mL”, Line 20; “5 mL”, Line 21; “5 mL”, Line 22; “5 mL”, Line 23; “5 mL”, Line 24; “5 mL”, and Line 25; “5 mL” were incorrect. These should have read: Line 3; “20 μL”, Line 5; “5 μL”, Line 20; “5 μL”, Line 21; “5 μL”, Line 22; “5 μL”, Line 23; “5 μL”, Line 24; “5 μL”, and Line 25; “5 μL”.

We apologize for these errors.

